# Integrating HIV Care into Primary Care Services: Quantifying Progress of an Intervention in South Africa

**DOI:** 10.1371/journal.pone.0054266

**Published:** 2013-01-22

**Authors:** Kerry E. Uebel, Gina Joubert, Edwin Wouters, Willie F. Mollentze, Dingie H. C. J. van Rensburg

**Affiliations:** 1 Department of Internal Medicine, Faculty of Health Sciences, University of the Free State, Bloemfontein, South Africa; 2 Department of Biostatistics, Faculty of Health Sciences, University of the Free State, Bloemfontein, South Africa; 3 Department of Sociology and Research Centre for Longitudinal and Life Course Studies, University of Antwerp, Belgium; 4 Centre for Health Systems Research and Development, University of the Free State, Bloemfontein, South Africa; Indiana University, United States of America

## Abstract

**Background:**

Integration of human immunodeficiency virus (HIV) care into primary care services is one strategy proposed to achieve universal access to antiretroviral treatment (ART) for HIV-positive patients in high burden countries. There is a need for controlled studies of programmes to integrate HIV care with details of the services being integrated.

**Methods:**

A semi-quantitative questionnaire was developed in consultation with clinic staff, tested for internal consistency using Cronbach's alpha coefficients and checked for inter-observer reliability. It was used to conduct four assessments of the integration of HIV care into referring primary care clinics (mainstreaming HIV) and into the work of all nurses within ART clinics (internal integration) and the integration of pre-ART and ART care during the Streamlining Tasks and Roles to Expand Treatment and Care for HIV (STRETCH) trial in South Africa. Mean total integration and four component integration scores at intervention and control clinics were compared using one way analysis of variance (ANOVA). Repeated measures ANOVA was used to analyse changes in scores during the trial.

**Results:**

Cronbach's alpha coefficients for total integration, pre-ART and ART integration and mainstreaming HIV and internal integration scores showed good internal consistency. Mean total integration, mainstreaming HIV and ART integration scores increased significantly at intervention clinics by the third assessment. Mean pre-ART integration scores were almost maximal at the first assessment and showed no further change. There was no change in mean internal integration score.

**Conclusion:**

The questionnaire developed in this study is a valid tool with potential for monitoring integration of HIV care in other settings. The STRETCH trial interventions resulted in increased integration of HIV care, particularly ART care, by providing HIV care at referring primary care clinics, but had no effect on integrating HIV care into the work of all nurses with the ART clinic.

## Introduction

There is international agreement that universal access to treatment for people with human immunodeficiency virus (HIV) in high-burden countries will not be achieved by vertical or single disease approaches to delivering HIV care, but rather by providing HIV care within general health systems [1], [2], [3]. Calls have been made to use international funding and support for HIV care to strengthen general health systems, and broaden existing vertical HIV programmes so as to provide HIV care within general health systems – the so-called diagonal approach [4], [5].Various strategies have been used in order to do this in countries with severe human resource shortages and struggling health care systems. These include shifting tasks from highly skilled to lower skilled and even lay health workers, mobilising community support and integrating HIV care into primary care services [3]. There are many reports of the effectiveness of task shifting [6], [7], [8], [9], [10], [11], and community mobilisation [12], [13] and guidelines have been published by the World Health Organization [14]. However, there are few clear recommendations on effective strategies to integrate HIV care into primary care services partly because there is still little evidence that integration of health care programmes does improve patient outcomes [15].

One of the problems is that integration is a broad concept. It has been defined as “a variety of managerial or operational changes to health systems to bring together inputs, delivery, management and organisation of particular service functions” [16].Integration may take place in all or any combination of a number of different health system functions including service delivery, management, financing, governance and monitoring and evaluation [17]. In order to provide evidence of the effectiveness of integration, controlled studies of integration are urgently needed. Such studies would need to describe what functions are being integrated and what strategies are used, but would also need to develop tools to monitor and quantify integration in order to correlate integration with outcomes achieved and compare outcomes across different studies [18].

There have been reports of strategies to integrate HIV care into primary care services including: co-location of vertically run HIV services in primary care facilities [19] down referral of stable patients on antiretroviral treatment (ART) to primary care clinics [20] and the provision of outreach support to primary care clinics from existing ART sites [21]. Other programmes have reported on strategies to integrate HIV care into all primary care consultations. These included staff training, standardised protocols, combined medical records and waiting areas, and the inclusion of HIV testing into triage [22], [23]. There are reports of improved access to ART with primary care driven models of HIV care [8], [21], [24], [25]. However all of these reports were observational and none were able to link patient outcomes with the extent to which HIV care was integrated.

This paper describes the development of a questionnaire as a tool to quantify integration of HIV care into primary care services achieved during a controlled trial of a complex intervention in the Free State Province of South Africa. This was a trial of a task shifting and integration intervention, monitoring the outcome of patients needing ART, called Streamlining Tasks and Roles to Expand Treatment and Care for HIV (STRETCH) [26]. The STRETCH trial is registered at isrctn.org (ISRCTN46836853)

## Methods

### Ethics statement

Approval to conduct this study was obtained from the head of the Department of Health in the Free State. The protocol for this sub-study was approved by the ethics committee of the Faculty of Health Sciences of the University of the Free State. The main STRETCH trial protocol was approved by the ethics committees of the faculties of health sciences at the Universities of Cape Town and the Free State. Clinic managers provided written informed consent to take part in the trial. As the STRETCH trial was an educational and managerial intervention aimed at entire clinics and their staff, all patients in the intervention clinics would be exposed to the same intervention. Informed consent was not requested from individual patients. Patients in intervention clinics were given written information about the trial. Ethical principles for use of medical records for research without patients' consent were followed: the research had a clear public benefit, approval was obtained for the study from the lead doctors and nurses managing the programme, use of the data for research did not influence individuals' care, the data were already being used by the research team for programme evaluation on behalf of the provincial health department, and data confidentiality was strictly enforced. Only selected data managers had access to personal identifiers. Anonymised data were provided only to the principal investigators, the lead statistician and the health economist. This consent procedure was approved by both ethics committees.

### Context of the study

The Free State province, with a population of 2.8 million [27], and an estimated HIV prevalence of 18.5% among 15–49 year olds [28], commenced the public sector rollout of ART in 2004 with a vertical approach to delivery of HIV care. Patients diagnosed as HIV-positive, by nurses from primary care clinics (who diagnose and treat common conditions) were referred for all further HIV care to designated ART nurses (also primary care nurses but appointed for the ART programme) at ART assessment sites located within selected primary care clinics. Patients eligible for ART (CD4 <200 cells/µl or Stage 4) had baseline bloods taken, received drug readiness training and then were referred for initiation of ART to doctors at ART treatment sites and subsequently fetched monthly supplies of ART at the assessment site. Those not yet eligible for ART continued to access care (CD4 counts through laboratory testing, staging, tuberculosis (TB) screening and cotrimoxazole prophylaxis) with ART nurses at the assessment site. By the end of 2007, 57 ART assessment and treatment sites were functioning [29]. However, the vast majority of primary care clinics in the province could not provide on-site access to HIV care but rather had to refer their patients to primary care clinics with ART assessment facilities. While patients on ART had good outcomes [30], [31], estimated coverage was only 25% [29], [32], the mortality rate amongst patients awaiting ART was high [30], and high rates of burnout in nurses working in ART clinics were recorded [33].

In order to assess strategies to improve access to ART, a pragmatic, cluster, randomised controlled trial, the STRETCH trial was conducted. All 31 existing ART assessment sites at the end of 2006, were randomised into 16 intervention and 15 control clinics within 9 clusters of between 2–7 clinics. Clinics in a cluster were usually under one local area or district management structure, or referred patients to doctors at the same ART treatment clinic, or both. The trial comprised two interventions: 1) nurse initiation and repeat prescription of ART and 2) integration of HIV care into primary care. The primary outcomes were survival of patients with CD4<350 and not yet on ART (patients eligible for ART or likely to become eligible during the trial) and 12 month viral load suppression rates for patients on ART [26], [34].

The integration intervention was developed in consultation with staff at all 31 clinics [34]. They reported that the existing system of ART nurses providing all HIV care at designated ART assessment sites was overloading these ART nurses and was also cumbersome for patients. One example of this was that HIV-positive patients on ART and those not yet eligible for ART who needed cotrimoxazole prophylaxis, accessed HIV care from ART nurses at the ART assessment site but had to fetch cotrimoxazole from primary care nurses at their local primary care clinic as cotrimoxazole was supplied from the clinic's primary care budget. The aims of the intervention developed with the staff were twofold: 1) HIV care was to be integrated into the work of all primary care nurses (and not just the ART nurses) within the ART clinic so that patients could access HIV care from any nurse at that clinic (internal integration) and, 2) HIV care was to be provided by nurses at all surrounding primary care clinics referring patients to that ART clinic, so that patients could access HIV care from their local clinic (mainstreaming HIV care). Staff also identified six elements of HIV care that needed to be integrated: 1) voluntary counselling and testing (VCT); 2) initial CD4 count; and 3) routine care including cotrimoxazole prophylaxis for those not yet eligible for ART (three elements of pre-ART care); 4) baseline blood tests; 5) drug readiness training; and 6) monthly supply of ART for patients eligible for ART (three elements of ART care). It was noted during development of the intervention that integration of pre-ART care had already commenced at some ART clinics and their surrounding primary care clinics (“referring primary care clinics”). ART prescription and adherence counselling could not be integrated into all primary care services during the trial as provincial authorisation of nurse ART prescription was limited to trained nurses in intervention clinics only.

The strategies used to implement the STRETCH interventions have been described elsewhere [34] and are summarised in Table 1. These included provincial training in diagnosis and care of TB, respiratory disease, sexually transmitted diseases and HIV at all primary care clinics using the PALSA PLUS primary care guidelines [35], [36], and extra training in ART prescription at intervention clinics using a STRETCH edition of PALSA PLUS. Clinic based teams, consisting of key clinic staff, implemented changes within intervention clinics. Local management teams comprising the local area manager, ART pharmacist and PALSA PLUS trainer, and clinic managers from the intervention clinic and referring primary care clinics, supported mainstreaming of HIV care. The STRETCH coordinator (KU) provided clinical and organisational support and was involved in facilitating the management teams. An implementation toolkit with descriptions of the trial interventions and changing roles of clinic staff, was distributed to members of the clinic based teams. The intervention was implemented in three phases at a pace decided by clinic teams.

**Table 1:** Intervention and control clinic characteristics during STRETCH trial.

	Intervention ART clinics	Primary care clinics referring to intervention clinic	Control ART clinics	Primary care clinics referring to control clinic
Nurse training	6–8 sessions of PALSA PLUS training. Extra 4 sessions STRETCH training in initiating and monitoring adults on ART	6–8 sessions of PALSA PLUS training	6–8 sessions of PALSA PLUS training	6–8 sessions of PALSA PLUS training
Provincial authorisation of ART prescription	Trained professional nurses authorised to initiate and repeat prescriptions of ART for uncomplicated adults	Patients referred to intervention site for ART prescription	Patients referred to doctors at treatment clinics for ART prescription	Patients referred to control clinics and thence to doctors at treatment clinics for ART prescription
Patient management guidelines	STRETCH edition of PALSA PLUS including guidelines for initiation and repeat prescription of ART	Standard Free State edition of PALSA PLUS guidelines	Standard Free State edition of PALSA PLUS guidelines	Standard Free State edition of PALSA PLUS guidelines
Implementation toolkit	STRETCH toolkit issued to members of clinic based team			
Clinic based support team	Clinic based STRETCH team to implement integration of pre-ART and ART care into work of all professional nurses in clinic			
Local area management support team		Local area management team to implement integration of pre_ART and ART care into all primary care clinics referring to intervention clinic		

Elements of STRETCH trial intervention including nurse training, patient care guidelines, toolkit and support teams at intervention clinics and their referring primary care clinics compared to standard care at control clinics and their referring primary care clinics.

### Integration questionnaire

Although provincial tools existed to monitor provision of HIV care (such as HIV tests and CD4 counts) at primary care clinics, there was no tool to assess whether HIV care was integrated into all consultations within clinics. Thus, a new questionnaire was developed in consultation with clinic staff (see Additional File S1 and a summary in Table 2). There were eleven questions on internal integration. These questions assessed the integration of care for HIV-positive patients into the consultations of all nurses within the ART clinic. There were four questions on the integration of HIV care for patients not yet eligible for ART (pre-ART care) (Q1,3,5 and 7); four on the integration of HIV care for patients eligible for and on ART (ART care) (Q12,14,16 and 18) and three questions on the integration of primary care services needed by patients on ART at that clinic (TB diagnosis, dispensing of cotrimoxazole and contraception) (Q9,10 and 11). There were eight questions on mainstreaming HIV care. These questions assessed the provision of HIV care by nurses at referring primary care clinics. There were four questions each on the provision of pre-ART (Q2,4,6 and 8) and ART care (Q13,15,17 and 19).

**Table 2:** Component questions of the five different integration scores.

Integration score	Component questions contributing to score	Example question
Total integration score	All 19 questions	
Pre-ART integration score	Q1–8 on the provision of HIV care for patients not yet eligible for ART by 1)all nurses within the ART site and 2) the patients local referring primary care clinic	Q4. If a patient is diagnosed HIV-positive at one of your referring PHC clinics is it possible to access their initial CD4 count at that clinic?
ART integration score	Q12–19 on the provision of HIV care for patients eligible for ART by 1)all nurses within the ART site and 2) the patients local referring primary care clinic	Q14. When patients from your clinic are about to start ARVs and need Baseline bloods who takes these bloods?
Mainstreaming HIV score	Q2,4,6,8,13,15,17,19 on the provision of pre-ART and ART care by the patients local referring primary care clinic	Q19. Is it possible for patients from your referring PHC clinics who are on ARVs to fetch their repeat supply of ARVs from their own PHC clinic?
Internal integration	Q1,3,5,7,9,10,11,12,14,16,18 on the provision of pre-ART and ART care by all nurses within the ART clinic *and* the provision of three key primary care services for patients on ART	Q1. If a patient needs an HIV test at your clinic who is performing this test?

A summary describing which questions from the integration questionnaire contributed to each integration score during the four assessments of the trial. An example of the questions contributing to each integration score is also included. The full questionnaire is included in Additional File 1.

Based on the initial discussions with staff, each question had only two or three possible responses to describe integration. Answers were scored 0 for no integration, 2 for full integration and, in questions with three responses, 1 for partial integration. The scores for each question were combined to give a total integration score and four component integration scores. These different combinations of questions and the resulting integration scores are summarised in Table 2 and described below:

- *Total integration score* – total score for all 19 questions.
- *Pre-ART integration score* – total score for questions 1–8 on the provision of HIV care for patients *not yet eligible for ART* by all nurses (primary care and ART nurses) at the ART clinic *and* at referring primary care clinics
- *ART integration score* – total score for questions 12–19 on the provision of HIV care for patients *eligible for, and on ART* by all nurses (primary care and ART nurses) at the ART clinic *and* at referring primary care clinics
- *Internal integration score* – total score for questions 1,3,5,7,9,10,11,12,14,16 and 18 on the provision by all nurses within the ART clinic, of pre-ART and ART care and on the provision of three key primary care services for patients on ART
- *Mainstreaming HIV score* – total score for questions 2,4,6,8,13,15,17,and 19 on the provision of pre-ART and ART care by nurses at referring primary care clinics

Internal integration scores could be calculated for all 31 clinics throughout the trial. However, the other integration scores could only be calculated for 23 clinics (13 intervention and 10 control) that still had primary care clinics referring patients to that ART clinic throughout the trial. New ART assessment clinics were established by the Department of Health during the trial and consequently eight of the 31 trial clinics no longer had patients referred from other primary care clinics by the last assessment of the trial.

The questionnaire was administered at all four assessments by the trial coordinator (KU) with the clinic manager or senior ART nurse at the clinic and preferably the same person at each assessment, but this was not always possible. The answer that best described the level of integration was decided by the interviewee in discussion with the coordinator. The coordinator was involved in local management teams responsible for the implementation of integration and so had independent confirmation about the progress of integration in each clinic. The assessments were done at all 31 clinics, as it could not be assumed that integration would not take place at control clinics [37]. Integration of HIV care into primary care services at the 16 intervention clinics commenced in Phase 1 of the trial and six-monthly integration assessments were planned. The first two assessments were conducted six months apart during early trial support visits. The last two assessments, when support visits were less frequent, were conducted telephonically, and the interval was extended to nine months as the trial had been extended due to a delay in nurse initiation of ART in some clinics. Time taken to complete the questionnaire was short (10–15 minutes), but as the coordinator had to travel to each clinic, or phone the interviewees at a time convenient to conduct the questionnaire, each round of assessments took four to six weeks to complete. A mean date of assessment was assigned in order to plot changes in mean scores.

### Consistency and reliability of the questionnaire

Internal consistency of the questionnaire was tested using Cronbach's alpha coefficients. These were calculated from scores at the first assessment for the entire questionnaire, and then the groups of questions on internal integration and mainstreaming HIV care, as well as the questions on pre-ART and ART integration.

In order to test for inter-observer reliability, the interview was repeated by a different interviewer at five clinics (three intervention and two control) in two districts, two months after the first assessments. These clinics were chosen by convenient sampling from the 23 clinics that had primary care clinics referring patients throughout the trial and could give scores for all 19 questions.

### Statistical analysis

Differences in the mean values of total integration scores and in the four component integration scores at intervention and control clinics were analysed with one way analysis of variance ANOVA (SPSS version 16.0) and a non-parametric analysis, Mann-Whitney (SAS version 9.2). Repeated measures ANOVA was used to analyse changes in mean scores over time (SPSS version 16.0). The level of significance was chosen as a p value of <0.05.

## Results

### Internal consistency and reliability of the questionnaire

Cronbach's alpha coefficient was 0.85 for all 19 questions, 0.86 for the 8 questions on pre-ART integration, 0.68 for the 8 questions on ART integration, 0.73 for the eleven questions on internal integration and 0.69 for the eight questions on mainstreaming of HIV care.

The second observer, who conducted repeat interviews at 5 clinics, obtained the same integration score on all questions at two clinics, a one point difference on one question only at two other clinics and a total score of one point difference with three questions scoring differently at the fifth clinic. The mean total integration score was 23.5 (maximum possible score 38) for the two assessments at the 5 sampled clinics by both observers. The mean difference between the total integration scores at the 5 clinics done by the two different observers was −0.6, with a standard deviation of 0.55 giving 95% limits of agreement of −1.7 to 0.5.

### Progress of integration

In an initial analysis of the changes in scores across all clinics for individual questions, the four questions that showed the largest absolute increases in integration scores (an increase of between 14–16 points) between the first and fourth assessments were questions 13,15,17 and 19 – all questions dealing with the mainstreaming of ART care. The questions that showed minimal variation in integration scores (absolute changes between 1–3 points) were questions 1–8 on mainstreaming and internal integration of pre-ART care.

**Figure 1:**
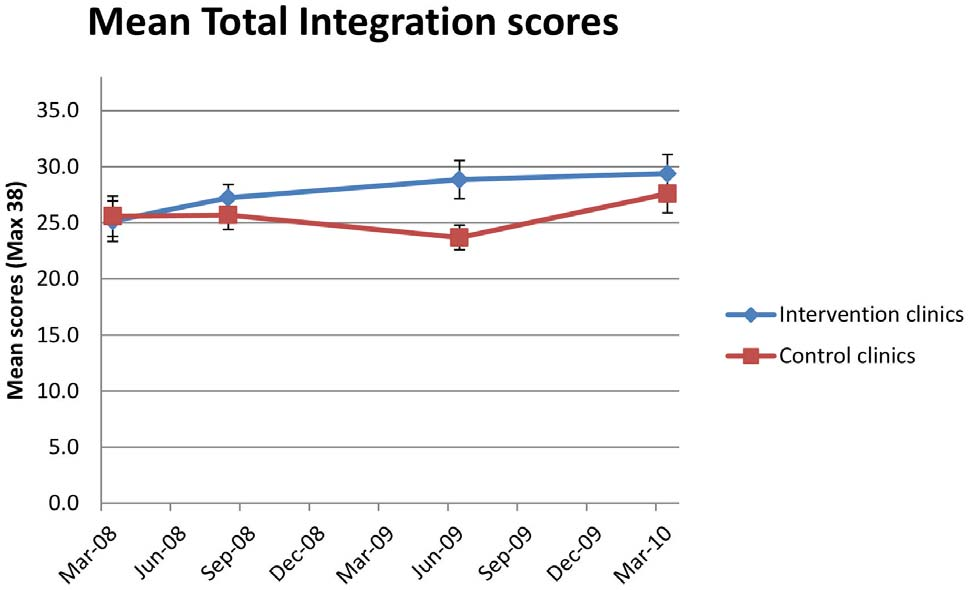
Progress of mean total integration scores during the STRETCH trial. Line graph of mean total integration scores at intervention and control clinics plotted against mean date of assessment, for four assessments during the STRETCH trail. Error bars depict standard error on the mean at each assessment.

**Figure 2:**
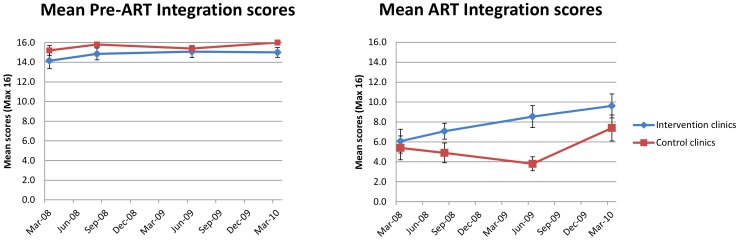
Progress of mean pre-ART and ART integration scores during the STRETCH trial. Two line graphs of mean pre-ART and ART integration scores at intervention and control clinics plotted against mean date of assessment, for four assessments during the STRETCH trial. Error bars depict standard error on the mean at each assessment.

**Figure 3:**
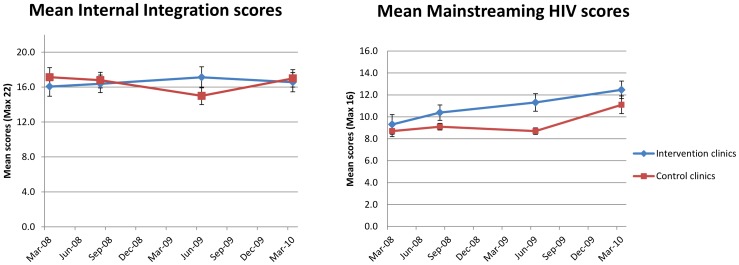
Progress of mean internal integration and mainstreaming HIV scores during the STRETCH trial. Two line graphs of mean internal integration and mainstreaming HIV scores at intervention and control clinics plotted against mean date of assessment, for four assessments during the STRETCH trial. Error bars depict standard error on the mean at each assessment.

The changes in mean integration scores for intervention and control clinics are plotted in Figures 1, 2, 3. As seen in Figure 1, mean total integration scores at the first assessment at intervention (25.7) and control clinics (25.4) were not significantly different. At the third assessment, conducted in the middle of the trial, the mean total integration score at intervention clinics (28.8) was significantly higher than at control clinics (23.7) (ANOVA, p = 0.0174; Mann-Whitney, p = 0.0267). The increase in mean total integration score at intervention clinics from the first (25.7) to the third assessment (28.8) was a significant change (rmANOVA, p = 0.0198). There was also a significant increase in mean total integration scores at control clinics that occurred late in the trial between the third (23.7) and fourth assessments (27.6) (rmANOVA, p = 0.0283). Consequently at the fourth assessment, there was no longer a significant difference between mean total integration scores at intervention and control clinics (ANOVA, p = 0.4581; Mann-Whitney, p = 0.5342).

In order to determine whether there was any change in which elements of HIV care had been integrated, differences in mean pre-ART and ART integration scores at intervention and control clinics and changes in these scores were analysed (see Figure 2). Mean pre-ART and ART integration scores at the first assessment at intervention clinics (14.4 and 6.2 respectively) and control clinics (15.4 and 5.1 respectively) were not significantly different. At the third assessment only, the mean ART integration score at intervention clinics (8.5) was significantly higher than at control clinics (3.8) (ANOVA, p = 0.0015; Mann-Whitney, p = 0.0029) and was significantly higher than at the first assessment (6.2) (rmANOVA, p = 0.004). However the mean pre-ART integration scores at intervention clinics were not significantly different from control clinics at any assessment, and there was no significant change at intervention or control clinics from the first assessment to the last assessment. Thus, integration of elements of ART care was the main contributor to the increased total integration scores at intervention clinics during the trial. The increase in mean total integration scores at control clinics late in the trial was likewise due to a significant increase in mean ART scores between the third (3.8) and fourth assessment (7.4) (rmANOVA, p = 0.0078). It was noted, however, that mean pre-ART integration scores were already close to the maximum possible score of 16 at the beginning of the trial and remained there throughout the trial.

In order to determine whether any significant change in integration at the two levels of primary care had taken place during the trial, differences in mean mainstreaming HIV and internal integration scores and changes in these scores were analysed (see Figure 3). Mean mainstreaming HIV and internal integration scores at the first assessment at intervention (9.7 and 16.1 respectively) and control clinics (8.8 and 17.1 respectively) were not significantly different. At the third assessment only, the mean mainstreaming HIV score at intervention clinics (11.3) was significantly higher than at control clinics (8.7) (ANOVA, p = 0.0073; Mann-Whitney, p = 0.0158) and significantly higher than at the first assessment (9.7) (rmANOVA, p = 0.0023). There were no significant differences in mean internal integration scores between intervention and control clinics at any assessment, and no significant changes in internal integration scores at intervention or control clinics from the first assessment to the last assessment. Mainstreaming of HIV care into primary care clinics was thus the main contributor to the level at which integration of HIV care into primary care took place at intervention clinics during the trial. The increase in mean total integration scores, late in the trial at control clinics, was also due to a significant increase in mean mainstreaming HIV scores occurring between the third (8.7) and fourth assessments (11.1) (rmANOVA, p = 0.0059).

## Discussion

This assessment shows that the strategies employed during the STRETCH trial resulted in significant increases in total integration scores at intervention clinics. The specific areas in which the integration score increased were in providing HIV care in primary care clinics not previously involved in the ART programme (mainstreaming HIV score) and in the provision of elements of ART care, namely, the taking of baseline blood tests, drug readiness training and monthly supply of ART for patients eligible for ART (ART score). These findings have been independently confirmed by a qualitative process evaluation of the STRETCH trial which found that patients and nurses appreciated the convenience of patients being able to access HIV care including ARVs at their local clinic instead of having to travel to an ART clinic [38]. There was no increase in mean pre-ART integration scores during the trial because these elements of HIV care, namely VCT, initial CD4 count and routine care for those not yet eligible for ART which had been identified by staff as critical elements of pre-ART needing integration, had already been substantially integrated into primary care services by local managers in the months leading up to the trial.

In contrast it appears that the strategies used in the STRETCH trial had no effect on internal integration scores at intervention clinics, with no significant shift towards patients being able to access HIV care from all nurses within the clinic. There may be other more effective strategies to achieve integration of HIV care into the work of all nurses within primary care clinics, or there may be factors that mitigate against internal integration. Topp et al described some strategies to integrate the provision of HIV care into the work of all nurses within two primary care clinics in Zambia [22]. These strategies included training of all staff in HIV care, as in the STRETCH trial, but also the use of other strategies not used in the STRETCH trial – combined medical records and waiting areas and the inclusion of HIV testing into triage of all patients. They did document increased uptake of HIV testing and good standards of HIV care. However, they also reported resistance on the part of nurses and patients to completely integrated ART services because of issues such as increased waiting times and the loss of informal support for patients on ART with the loss of ART waiting areas [22]. A synthesis of the findings of three qualitative studies on internal integration in Free State clinics conducted at the same time as the STRETCH trial found that administrative issues and patient and nurse preferences tended to mitigate against internal integration of HIV care (manuscript submitted for publication).

The increase in mean total integration, ARV and mainstreaming HIV scores by the fourth assessment late in the trial at control clinics, resulted from provincial implementation of a new national AIDS policy including nurse prescription of ART and the provision of ART in all primary care clinics – the two main interventions of the trial [39]. The STRETCH trial was a pragmatic trial conducted under real conditions which include such policy changes. The research team was able to negotiate with the province that nurse initiation of ART would not be implemented in control clinics till after the trial, but was not able to delay integration of HIV care into primary care services in control clinics in the last few months of the trial.

One of the strengths of this study is that it is a prospective assessment using a new semi- quantitative tool to document integration of HIV care. The contents of this questionnaire were likely to be valid as the elements of HIV care and the need to integrate them at both levels were identified in consultation with staff at ART clinics. Internal consistency as shown by Cronbach's alpha was good. Real validity of the questionnaire was demonstrated in that it captured an increase in integration scores at control clinics as a result of the implementation of a new policy to integrate HIV care into primary care in the last months of the trial.

There were some potential limitations to this study. The first two interviews were conducted during clinic visits while the last two were conducted telephonically, interviews were not always conducted with the same staff member and data on services at referring primary care clinics were based on reports from staff at the ART site and not at the primary care clinic. However, all interviews were conducted by the trial coordinator who was well known to the clinic staff and involved in local management teams implementing integration and thus was able to independently confirm progress of integration as described by the interviewee at each clinic. Though there is a possibility that the coordinator may have influenced answers, the results of inter-observer reliability tests suggest that this was negligible. The lack of progress in internal integration compared with the progress in mainstreaming HIV, captured by the questionnaire, suggests that the interviewees were not unduly influenced to report integration where there was none. The integration questionnaire was developed to assess the integration of HIV care into primary care as it affected service delivery for patients and was therefore not able to assess the effects of integration of other areas of health system functioning. The questionnaire was not able to document the impact of integration of HIV care on the provision of other primary care services. This is an important area of research, and is the subject of a project currently being conducted in all primary care clinics in the Free State.

This questionnaire was validated in the specific context of the Free State and may need some further development, but it could be a valuable tool for assessing integration of HIV care into primary care clinics in other settings. The main results of the STRETCH trial showed that patient survival was not significantly different in intervention clinics compared with control clinics [40].The integration scores obtained in this study will be correlated with survival of patients with CD4 below 350 and not yet on ART, from the STRETCH trial to determine if integration of HIV care may have had an independent effect on patient survival. These results, together with the process evaluation and results of the STRETCH study, should be useful in identifying whether integration is an effective strategy to improve survival of HIV-positive patients in need of ART.

## Conclusion

The integration questionnaire developed in this study is a valid tool with potential to monitor integration in other high HIV-burden countries. This study demonstrated an increase in total integration scores in clinics in the Free State province during the STRETCH trial. This was achieved by integrating ART care, particularly at primary care clinics not previously designated as ART clinics but there was no increase in integration of HIV care into all consultations. The scores documented in this intervention will be used to determine if integration is associated with an improvement in survival of patients needing ART.

## Supporting Information

File S1 **Survey of integration of HIV care in ARV clinics and their referring primary care clinics.**
(DOC)
