# Supplementary material for: Integrating HIV Care into Primary Care Services: Quantifying Progress of an Intervention in South Africa
Source: PLoS One. 2013 Jan 22;8(1):e54266. doi: 10.1371/journal.pone.0054266 (PMC3551935; doi:10.1371/journal.pone.0054266)
Supplement: File S1 — Survey of integration of HIV care in ARV clinics and their referring primary care clinics. (DOC) [file pone.0054266.s001.doc]

**File S1.**

**Survey of integration of HIV care in ARV clinics and their referring PHC clinics**

Clinic and personal information

Name of clinic_____________________________________________

Town/city____________________ District______________________

Date of interview___________________________________________

Interviewer________________________________________________

Job description of interviewee_________________________________

Number of Professional nurses at clinic_________________________

Number of staff nurses at clinic________________________________

Number of enrolled nurse assistants at clinic_____________________

Number of ARV professional nurses at clinic____________________

Number of ARV staff nurses at clinic___________________________

Number of lay counsellors____________________________________

Number of referring PHC clinics in same town___________________

Number of referring clinics in nearby towns_____________________

Number of mobile clinics from this clinic_______________________

Comments on clinic function__________________________________________________________________________________________________________________________________________________________________

Diagnosis staging and routine HIV care

1. If a patient needs an HIV test at your clinic who is performing this test?

- ARV nurses at your clinic only
- PHC and ARV nurses at your clinic

1. If a patient needs an HIV test at one of your referral PHC clinics

- None of your referring clinics can do an HIV test
- Some of your referring PHC clinics can do an HIV test
- Most of your referring PHC clinics can do an HIV test

1. At your clinic who does the initial CD4 count for a newly diagnosed HIV positive patient

- ARV nurses at your clinic only
- PHC and ARV nurses at your clinic

1. If a patient is diagnosed HIV positive at one of your referring PHC clinics is it possible to access their initial CD4 count at that clinic?

- None of your referring clinics
- Some of your referring PHC clinics
- Most of your referring PHC clinics

1. If a patient at your clinic needs routine HIV care i.e. 6 or 12 monthly CD4, Pap and HIV Staging (for people with CD4 >200 not yet needing ARVs), who provides such care?

- ARV nurses at your clinic only
- PHC and ARV nurses at your clinic

1. Is it possible for a patient from one of your referring PHC clinics to access routine HIV care at that clinic?

- None of your referring clinics
- Some of your referring PHC clinics
- Most of your referring PHC clinics

1. If a patient at your clinic is stage 3 HIV but does not yet need ARVs, who can provide them with Cotrimoxazole prophylaxis

- ARV nurses at your clinic only
- ARV and TB nurses at your clinic only
- PHC and ARV and TB nurses at your clinic

1. If a patient from one of your referring PHC clinics needs Cotrimoxazole prophylaxis is it possible for them to get it at the PHC clinic?

- None of your referring PHC clinics
- Some of your referring PHC clinics
- Most of your referring PHC clinics

1. If patient at your clinic has symptoms of TB, who does the TB investigation)

- TB nurses at your clinic only
- TB and ARV nurses only
- PHC TB and ARV nurses at your clinic

Patients enrolling and on followup on ARVS

1. If a patient on ARVS at your clinic comes to fetch ARVs and needs family planning who can give it to them

- Family Planning nurse only?
- Any nurse at your clinic?

1. If a patient on ARVs is also on Cotrimoxazole prophylaxis do they fetch it

- At the same place that they fetch their ARVs?
- At your clinic but on the PHC side?
- At their own PHC clinic?

1. When patients at your clinic need to go to Drug Readiness Training who does the training?

- ARV site nurses do all three sessions
- ARV site nurses with Lay counselors assisting

1. When patients from your referring PHC clinics need to go to Drug Readiness Training who does the training?

- None of your referring PHC clinics
- The nurses or counselors at some of your referring PHC clinics
- The nurses or counselors at all of your referring PHC clinics

1. When patients from your clinic are about to start ARVs and need Baseline bloods who takes these bloods?

- ARV nurses only
- PHC and ARV nurses at your clinic

1. When patients needing ARVs from your referring PHC clinics need baseline bloods who can take these bloods

- None of your referring PHC clinics
- Some of your referring PHC clinics
- Most of your referring PHC clinics

1. When patients from your clinic on ARVs come for monthly follow-up nurse visits, who can see them

- ARV nurses only
- PHC and ARV nurses at your clinic

1. When patients on ARVs from one of your referring PHC clinics, need to come for monthly follow-up nurse visits, can they go to their PHC clinic?

- None of your referring PHC clinics
- Some of your referring PHC clinics
- Most of your referring PHC clinics

1. Where do patients from your clinic fetch their repeat supply of ARVs

- From ARV nurse
- From ARV pharmacy
- From main pharmacy at your clinic

1. Is it possible for patients from your referring PHC clinics who are on ARVs to fetch their repeat supply of ARVs from their own PHC clinic?

- None of your referring PHC clinics
- Some of your referring PHC clinics
- Most of your referring PHC clinics
